# The deep origin of ground fissures in the Kenya Rift Valley

**DOI:** 10.1038/s41598-023-30918-z

**Published:** 2023-03-04

**Authors:** Zhijie Jia, Hongjie Wu, Jianbing Peng, Quanzhong Lu, Weiliang Huang, Chuntao Liu, Feiyong Wang, Yang Liu, Ming He

**Affiliations:** 1 School of Geological Engineering and Geomatics, Chang’an University, Xi’an, China; 2 School of Earth Science and Resources, China University of Geosciences, Beijing, China; 3 Key Laboratory of Western China Mineral Resources and Geological Engineering, Xi’an, China; 4 School of Earth Sciences, Zhejiang University, Hangzhou, China

**Keywords:** Natural hazards, Solid Earth sciences

## Abstract

Intense volcanic and geothermal activities characterize the Great Rift Valley of East Africa. Ground fissure disasters of the Great Rift Valley have garnered increasing attention in recent years. Through field investigations, trenching, geophysical exploration, gas sampling and analysis, we determined the distribution and origin of 22 ground fissures within the Kedong Basin of the Central Kenya Rift. These ground fissures caused varying degrees of damage to roads, culverts, railways, and communities. Trenching and geophysical exploration have shown that ground fissures in sediments are connected to rock fractures with gas escaping. The gases expelled from the rock fractures contained methane and SO_2_, which were absent in the normal atmosphere, and ^3^He/^4^He ratios in gases measured further indicated that the volatiles were derived from the mantle, suggesting that these rock fractures extended deep into the underlying bedrock. Spatial correlations with rock fractures demonstrate the deep origin of these ground fissures, which are associated with active rifting, plate separation, and volcanism. The ground fissures are formed due to movement on the deeper rock fractures, and then the gas escapes through the fissures. Determining the unusual origin of these ground fissures can not only guide infrastructure development and urban planning but also contribute to the safety of local communities.

## Introduction

Ground fissures, known as Earth fissures, are defined as linear fractures with openings or offsets arising from the land surface^1^. Ground fissures develop due to the influence of various geological and human activities, and can create disasters in populated areas^2^. In September 1927, the first modern ground fissure was discovered in Arizona, USA^3^. Since then, disasters caused by ground fissures have been widely reported worldwide, including in the USA^4–8^, China^9–16^, Southeast Asia^17,18^, India^19^, Mexico^20–22^, Saudi Arabia^23–25^ and some coastal countries^4^. Severe disasters caused by ground fissures disrupt stable geological structures, such as ground fissures in the Weihe Basin have damaged the integrity of the surface sediments over a wide range^26^; cause economic losses such as the ground fissures in New Zealand, western United States and the northern China^27–29^; and threaten the safety of communities and livestock by destroying infrastructure and buildings such as the ground fissures of Arizona in the United States and Shanxi Province in China^30,31^. At present, research on ground fissures mainly focuses on their origin, which can explain their occurrence.

Studies on the origin of ground fissures generally suggest that their formation is related to various internal and external geological forces, including earthquakes, fault activity, sediment properties, and land subsidence^32–34^. Ground fissures can originate from either deep or shallow areas (the "shallow" in this article generally means the depth of 0–40 m, and the "deep" means several kilometers depth and even reaches the lithospheric mantle at a depth of tens of kilometers). Ground fissures originating in the shallow subsurface are mainly related to land subsidence and rainfall or the deformation of an aquifer caused by pumping concentrates the stress in the sediment. Inhomogeneous deformation of aquifers leads to differential compaction of sediments. The magnitude of the tensile stress generated by this differential compaction is proportional to the change in thickness of the aquifer. Ground fissures can be formed when the tensile stress exceeded the tensile strength of the sediment^35–38^.

Another type of ground fissure with a shallow origin is related to the nature of sediments. Loose sediments in the basin are easily transported by surface runoff originating from heavy rainfall, creating fissures; this phenomenon is more pronounced in fine-grained sediments^39^. In the Loess Plateau of China, owing to the collapsibility of loess, ground fissures of shallow origin typically occur when the loess has been soaked and saturated^40,41^.

Ground fissures originating in deep areas are mainly related to faults. Displacements and strains in the crust caused by faulting can directly lead to ground fissures^42^. The creep of deep hidden faults destroys the structure of the crust and produces numerous tiny fractures. These fractures, as prototypes of ground fissures, gradually expand and extend upward under the action of various internal and external geological forces, and finally appear on the surface^43–45^.

This research focuses on ground fissures within the Kedong Basin of the Central Kenya Rift, which is a part of the east branch of the Great Rift Valley of East Africa. As the most famous continental rift on Earth, the Great Rift Valley of East Africa is characterized by lithospheric thinning and brittle crustal subsidence caused by the intrusion of the upper mantle asthenosphere^46^. The separation boundary between the Nubian, Somali, and Arabian plates appears as an independent tectonic basin spanning thousands of kilometers on the surface^46^. From the Afar triple junction point, from north to south, the east branch of the Great Valley Rift is divided into the Ethiopian Rift Valley, Turkana lowland, and Kenya Rift Valley (KRV). The Great Rift Valley is characterized by active magmatism, magmatic intrusions and extensive geothermal fluids^47–51^. Since the first ground fissures were reported at the Main Ethiopian Rift (MER), which is the northern part of the Great Rift Valley^52^, the number of disasters caused by ground fissures has been increasing. At present, research on ground fissures in the East African rift is still in its infancy, and the research area is mainly limited to the main Ethiopian rift. The origin of ground fissures in the Great Rift Valley is thought to be mainly related to the extension of the fault or pipping caused by rainfall^53–55^. In 2018, Daily Nation reported the first ground fissure of KRV, namely, a huge fissure that damaged a road and caused livestock loss in Mai Mahiu, a town west of Nairobi, Kenya^56^.

The main feature of the study area is that it contains three active Quaternary volcanoes and extensive rock fractures. Based on our field investigation of ground fissures in the KRV, we produced a distribution map of ground fissures, established the spatial structural relationship between ground fissures and rock fractures, and determined the origin of ground fissures through trenching, geophysical exploration, and sample testing. This study fills the gap in ground fissure research in the Great Rift Valley region of Kenya and contributes to improving the community development and safety in this region.

## Geologic setting

The eastern branch of the Great Rift Valley, including the main Ethiopian Rift and the Kenya Rift Valley, is dominated by peralkaline magma, representing extensional setting and hotspots^57–61^. Study area is a part of central Kenya Rift. The strike of the rift valley is roughly north–south. The height of the steep margins on both sides of the rift valley can reach 3000 m, whereas the elevation of the inner valley of the rift is approximately 1000 m. Active faults on the rift margins are mainly high-angle normal faults with NE and NW strikes. The inner rift basin contains with loose sediments of varying thicknesses at the land surface, and ground fissures are mainly developed in these sediments. Since the late Pleistocene, the depressions in the Kenya Rift Valley were filled with pyroclastics^62^. These pyroclastic rocks are light yellow, porous and loose, mainly cemented by volcanic debris, crystal debris and volcanic glass. Most of the pyroclastic rocks are currently covered by loose sediments several meters thick, but some are still exposed at the valley floor. Extensive volcanic activity in the Kenya Rift effectively filled the basin and resulted in a series of shallow, ephemeral lake basins^63^. These lake basins are widely distributed such that the rift floor is covered with lacustrine sediments (e.g., alluvial silt and diatomite)^64,65^. Some of these lakes are saline lakes, and the silt sediments on the edges of these saline lakes may have disrupted the original sedimentary structure due to bioturbation^66–68^. During the past 0.7 Ma, volcanic activity created a series of axial craters (such as Mt. Suswa and Mt. Longonot) in the Kedong Basin and deposited a lot of volcanic ash on the floor and west side^64^. In the last 0.4 Ma, the Kedong Basin was closed due to the growth of the Mt. Suswa, the sediments in the Kedong basin are dominated by lacustrine accompanied by alluvial rework volcanic ash^69^. Subsequently, a very large flood brought sand and gravel formed by bedrock erosion and grinding into the Kedong Basin^64,70^.

**Figure 1:**
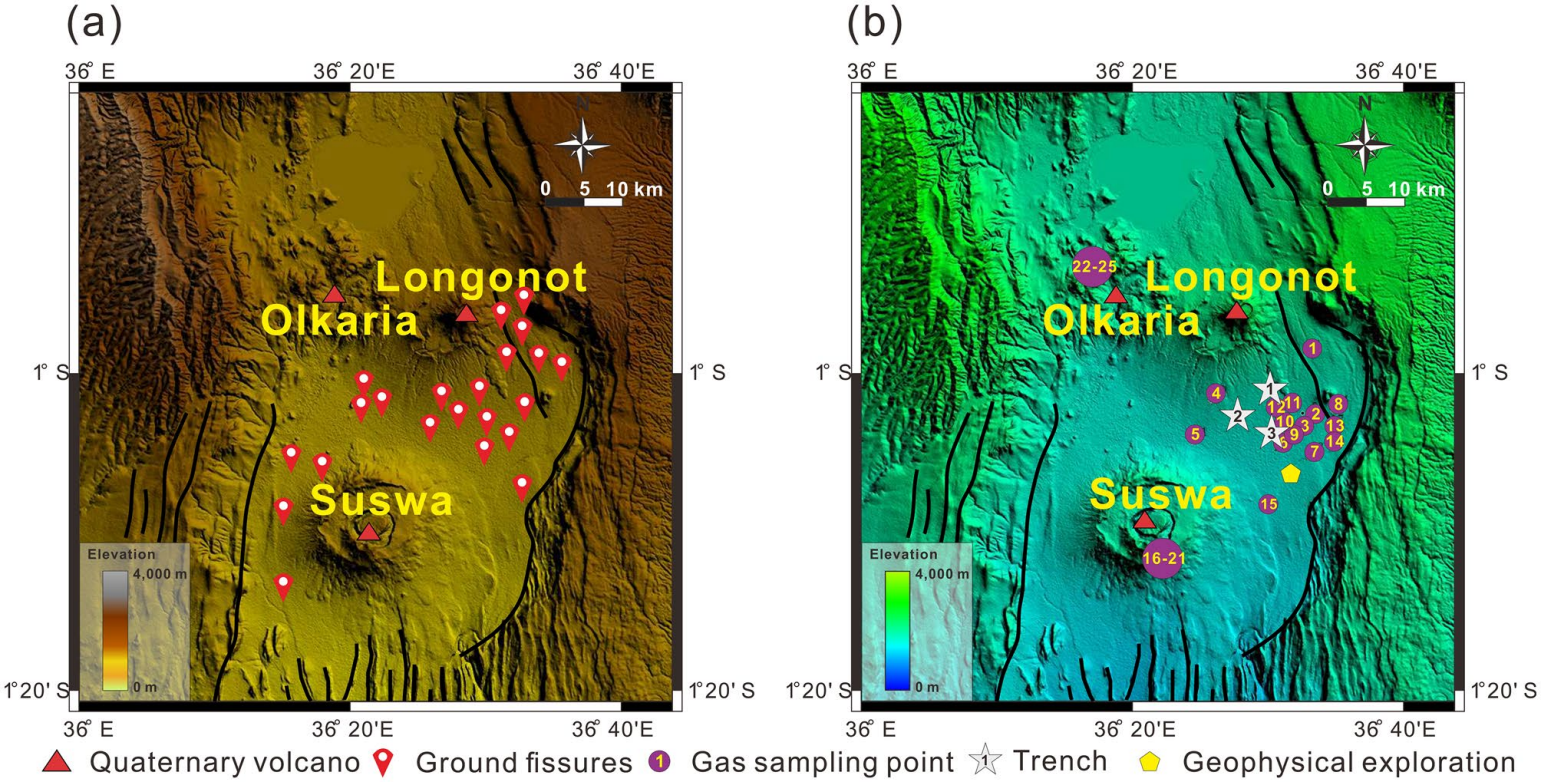
Map showing the (**a**) distribution of volcanoes and ground fissures, and the (**b**) location of gas samples, trenches, and geophysical analysis. The locations of No. 1, No. 2 and No. 3 trenches are the same as those of No. 12, No. 4 and No. 6 gas samples, respectively. The Digital Elevation Map (DEM) used is from Google-Earth, SRTM 90 m DEM Digital Elevation Database (https://srtm.csi.cgiar.org) and compiled in Global Mapper.

There are multiple volcanic craters of different sizes in the study area, the largest and most active of which are Mt. Suswa, Mt. Longonot, and Olkaria volcano (Fig. 1)^57^. These three Quaternary volcanoes are part of the central Kenya peralkaline province, Mt. Suswa, Mt. Longonot are trachytic caldera volcanoes, Olkaria is rhyolitic dome complexes. The products of these volcanoes are chemically different, so despite their proximity, they still indicate different magmatic systems^71^.

Mt. Suswa is a Quaternary, shield volcano composed of trachyte, phonolite lavas and tuffs^72,73^.Three main eruptions can be distinguished at Mt. Suswa: the first produced a primitive shield volcano; the second was accompanied by caldera formation; the third produced lava that partially filled the caldera^72,73^.Corresponding to the eruption history, the eruption products of Mt. Suswa can be divided into two parts, namely the lava and tuffs that formed the volcanic shield in the early stage and the lava and tuffs that erupted from the caldera later; these rocks are mostly peralkaline^74^. The pattern of the Mt. Suswa reflects both the main trend of the rift and the oblique northeast–south–west trend controlled by faults. Rock fractures are mainly distributed along the island block and there are no fractures or faults around Mt. Suswa, which may be due to the fact that these structures were concealed by later formation^65^. Mt. Longonot is a large Quaternary stratovolcano with a 12 km caldera^75^. The eruption of Longnot Volcano consists of seven episodes, dominated by peralkaline trachyte lavas, mixed Hawaii teperalkaline lava flows and pyroclastics^69,76^. Eruptive center and rock fractures of the volcano are controlled by NNW-trending boundary fault^77^. The isotopic composition of the fluids suggests that the geothermal reservoir of Mt. Longonot is recharged by atmospheric rainfall from the eastern rift shoulder, which may be distinct from recharge from Lake Naivasha to the north^78^. Olkaria is a late Quaternary to recent volcano, composed of multi-centred lava lava and dome filed, dominated by peralkaline rhyolites eruptions and the magma mixing occurs in all rock types^79,80^. The graben structures and faults control the fluid migration and the geothermal reservoirs of Olkaria^79^. Seismic monitoring data showed that the alignment of the epicenters matched the fault zone, which may reveal the migration of fluids along the fault^81^. Interferometric synthetic aperture radar (InSAR) measurements show that Mt. Suswa volcano sank by 2–5 cm during 1997–2000 and Mt. Longonot rose by 9 cm during 2004–2006^82^. The Mogi source model constrains the deflation center of the Mt. Suswa to a depth of 2.1–3.7 km, and the inflation center of the Mt. Longonot to a depth of 4 km^82,83^. Observations reveal active magmatic systems beneath these volcanoes, with migration patterns of geothermal fluids strongly dependent on rift structures and faults rather than volcanoes^82^.

Degassing from fumaroles in the Central Kenya Rift Valley is widespread, with initial studies showing that the vapors consisted mainly of atmospheric precipitation^65^. Atmospheric precipitation is heated and evaporated by rocks and returns to the surface in the form of fumaroles. The most important component is CO_2_, and the volatile substances in it are reasonably considered to be formed by the accumulation of residual volatiles in the magma chamber^65^. Further study found stable isotopes of CO_2_ from the rift flanks are mantle characteristic, indicating gas penetration in the faults, these faults may reach to deep and serve as major channels for CO_2_ into shallow hydrothermal systems^84^. Latest study shows that structure related to volcanism rather than regional tectonics controls fluid migration and degrassing in KRV, although these gases are obviously contaminated by the atmosphere, but carbon isotope indicate the mantle origin of these volatile materials^75^.

**Figure 2:**
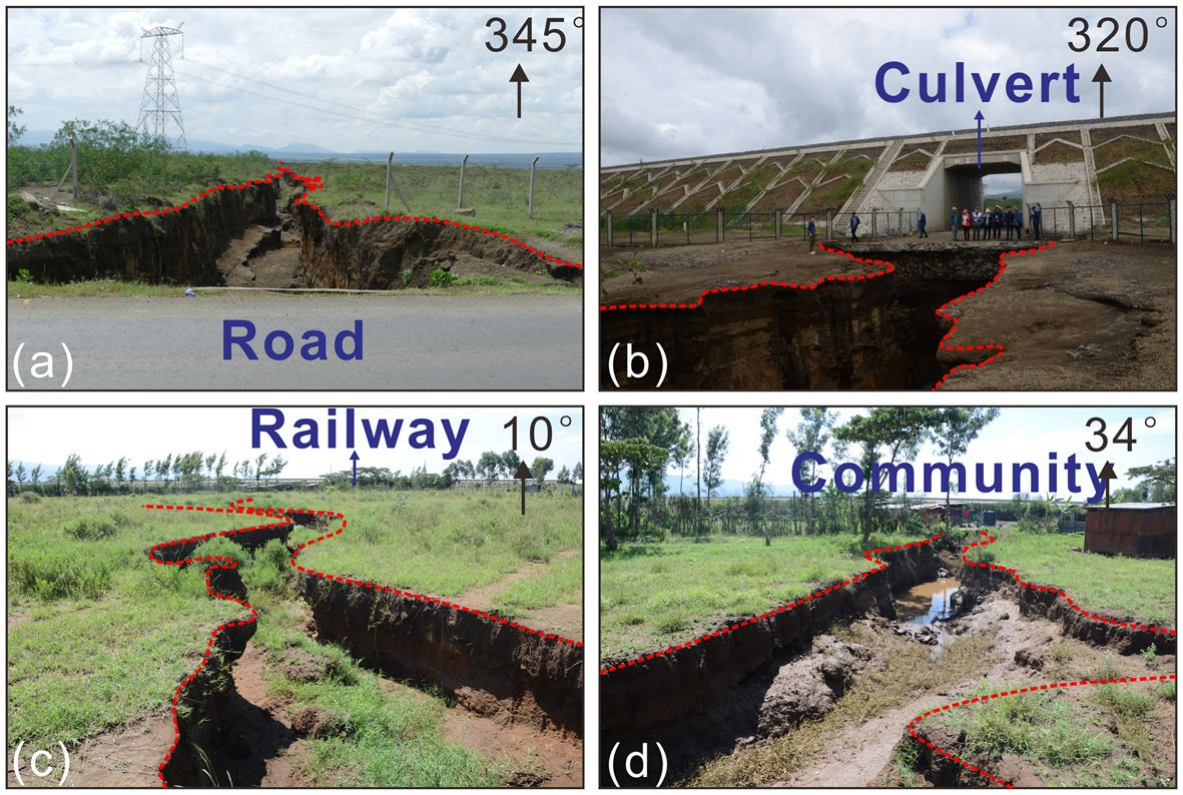
Photos showing the damage of ground fissures to a: (**a**) B3 road located in the flat area between Mt. Suswa and Mt. Longonot in the inner rift, (**b**) culvert located 500 m south of the B3 road shown in Fig. 2a, (**c**) railway located 800 m south of culvert shown in Fig. 2b, and (**d**) community located next to the railway shown in Fig. 2c.

A total of 22 ground fissure points was identified in the field survey, and the ground fissures mainly developed in the following three areas: around the active fault on the northeast side of the study area; the plain area between Mt. Longonot and Mt. Suswa; and the west side of Mt. Suswa (Fig. 1b). The shape of ground fissures is mainly linear, with a local “S” shape; the extension length varies greatly and there is no dominant strike. Ground fissures only show horizontal openings, and the two walls of the fissure are of the same height, with no shearing or offset (Fig. 2). The depth of the fissures is also inconsistent, with some fissures cutting through all sediment and reaching the bedrock, and most fissures being approximately 50 cm deep.

Ground fissure disasters threaten the safety of transportation facilities and communities in the study area. The ground fissures extend under the B3 road and gradually erode the subgrade, causing the foundation to settle and deform, eventually leading to the complete destruction of the road surface (Fig. 2a). The same mechanism also applies to the destruction of culverts (Fig. 2b) and railways (Fig. 2c) through ground fissures. The continuous extension of ground fissures not only directly damaged roads and railways in the study area but also restricted the siting of engineering facilities and traffic planning. The ground fissures that exist in a community also threaten the safety of its residents and livestock. According to local residents, there have been several reports of livestock falling into ground fissures. In some communities, the ground fissures are immediately next to the houses (Fig. 2d), and even go directly through the houses, which threatens the safety of residents. Additionally, ground fissures may provide channels for surface pollutants to descend into the aquifer and further contaminate groundwater.

## Results

A large number of rock fractures were present in the study area, and gas escaped from many of these fractures. In this section, we demonstrated the characteristics of rock fractures in the study area, their relationship with ground fissures, and the composition and origin of the gases escaping the fractures.

### Ground fissures and rock fractures

**Figure 3:**
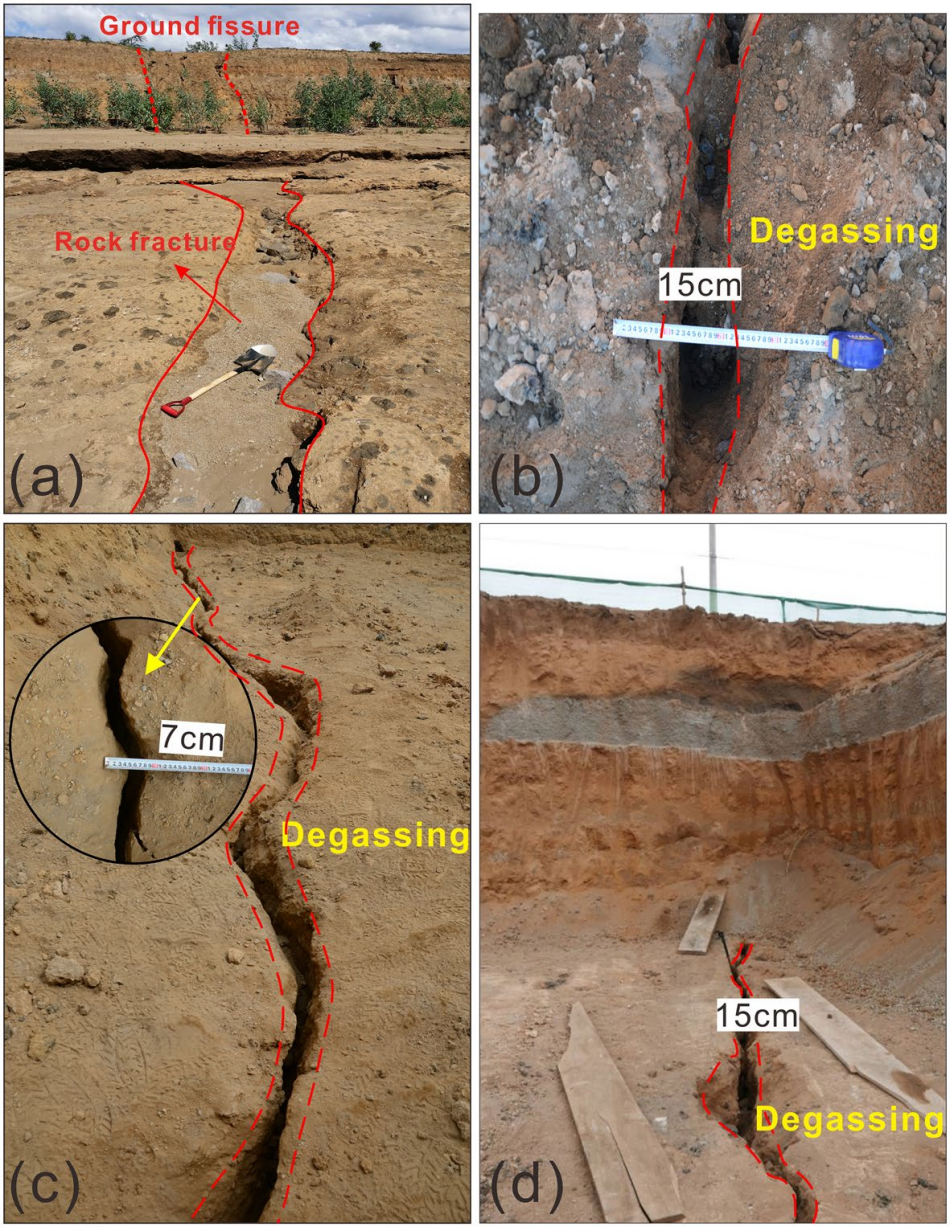
Spatial relationship between ground fissures and rock fractures. (**a**) Field investigations show that ground fissures are connected to rock fractures beneath sediment, and rock fractures have the same strike as ground fissures but have a narrower width; (**b**), (**c**) and (**d**) rock fractures with gas releasing revealed by trenching, these photos were taken in trenches No. 1, No. 2, and No. 3, respectively.

Unconsolidated sediments blanket the land surface of inner rift and loose pyroclastic rocks lie beneath several meters of sediments. In some gullies, pyroclastic rocks are directly exposed to the land surface. Extensive rock fractures are present on the surface of the pyroclastic rock, and the widths of these fractures vary from a few centimeters to 1 m. Field surveying and trenching (see “Methods” section) demonstrate that rock fractures occur under the ground fissures in the sediments, and the fractures have the same strike as the ground fissures, but are narrower in width than the ground fissures (Fig. 3a). To determine whether the spatial relationship between the rock fractures below the ground fissures is typical, we conducted trenching at three ground-fissure sites in the study area (see “Methods” section), the results show that rock fractures extended below each ground-fissure site (Fig. 3b–d). The rock fractures each correspond to the ground fissures above and have the same strike, but the widths of the fractures range from 7 to 15 cm, which are narrower than those of the ground fissures range from 0.5 to 4.8 m. The depths of the rock fractures were greater than the measurement range of the steel tape, which means the minimum depth also exceeds 25 m. The rupture characteristics of the rock fractures are consistent with those of ground fissures, showing openings without offset and shearing. Notably, gas escaped from each revealed rock fractures. The gas was warm and moist but had no smell.

### Shallow crustal structure

**Figure 4:**
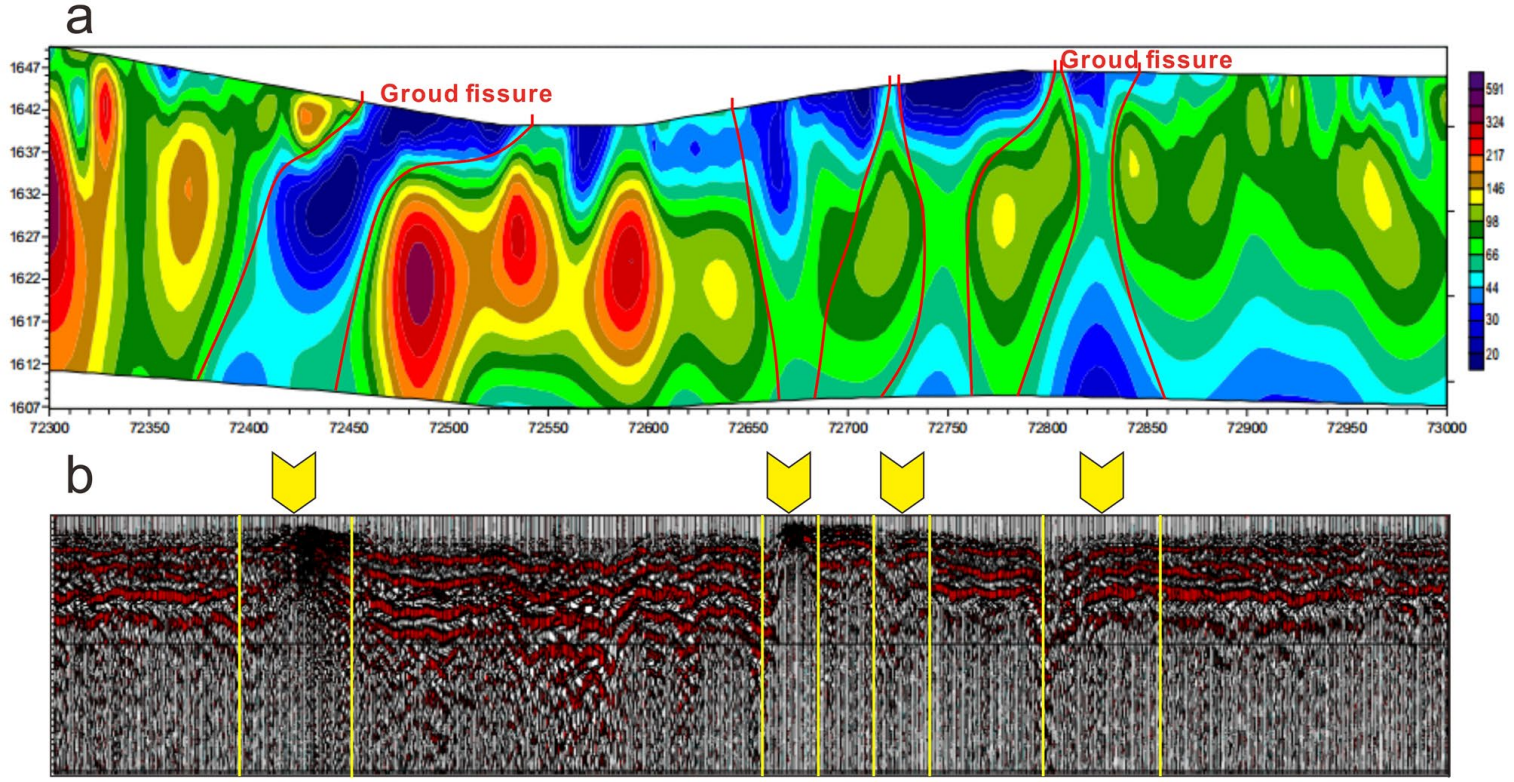
Geophysical characterization of ground fissures: (**a**) resistivity profile and (**b**) shallow seismic profile.

Using resistivity method and shallow seismic refraction method (see “Methods” section), we revealed the geophysical profile of a typical shallow crustal structure. Geophysical results showed that: the resistivity in the shallow crust (0–40 m) was generally low; the resistivity distribution of the overlying strata was uneven; and the seismic event was disordered. We inferred that the sedimentary stratum is relatively fragmented when compared to those areas without ground fissures (Fig. 4). The resistivity profile showed four distinct low-resistivity anomalies (Fig. 4a), two of which had ground fissures on the surface. Each low-resistance anomaly area corresponded to an obvious offset of seismic events in the shallow seismic profile (Fig. 4b). The results of the geophysical analysis further indicate the presence of a common ground fissure-rock fracture structure in the study area. This common structure consisted of ground fissures in the loose sediment and fractures in the underlying pyroclastic rock. Ground fissures and rock fractures represent the performance of the same geological structure in different formation. Trenching shows that the width of ground fissures in loose sediment is wider than that of the rock fractures in the underlying pyroclastic rocks. The results of geophysical measurements only reveal that the origin depth of the ground fissure is greater than 40 m, and more evidence is needed to further reveal its origin.

### Composition and origin of gases

The Great Rift Valley is characterized by intense volcanic and geothermal activity. The rock fractures in the study area exhibit a common degassing phenomenon. The study of the composition and origin of gases escaping the fractures can help us to obtain constraints on the depth of rock fractures and the origin of ground fissures. We collected 25 gas samples in the study area and analyzed their components and helium isotopes in the laboratory (see “Methods” section). The location of the gas samples is shown in Fig. 1b, and their latitude and longitude are shown in Table 2.

We collected 25 gas samples from the study area, the locations of which are shown in Fig. 1b. These gases originate from the emissions of rock fractures.

Table 1 presents the main components of the gas, which are oxygen (O_2_) and nitrogen (N_2_), accounting for approximately 99% of the total composition. Other conventional gas components also include carbon dioxide (CO_2_) and argon (Ar), of which CO_2_ and Ar account for approximately 0.03–0.09% and 1% of the total composition, respectively. The conventional compositional characteristics of the gas show an abnormally high value of CO_2_, and the CO_2_ concentration in the gas can be more than twice that in the atmosphere, assuming that the CO_2_ concentration in the atmosphere is approximately 0.03–0.04%. This, to some extent, implies that the gas has a different origin from the atmosphere.

**Table 1:** Composition of gas samples in the study area.

Number	Lat	Long	CO_2_ (%)	N_2_ (%)	O_2_ (%)	Ar (%)	CH_4_ (%)	SO_2_ (%)
1	− 0.955	36.545	0.025	79.76	19.23	0.98	0.0020	0.00006
2	− 1.02	36.538	0.065	79.26	19.72	0.95	0.0012	0.00006
3	− 1.041	36.533	0.052	79.30	19.69	0.95	0.0022	0.00009
4	− 1.009	36.430	0.068	78.47	20.43	1.03	0.0009	0.00002
5	− 1.051	36.411	0.077	79.35	19.61	0.96	0.0007	0.00007
6	− 1.055	36.509	0.091	79.25	19.72	0.93	0.0005	0.00006
7	− 1.07	36.539	0.089	79.27	19.67	0.97	0.0003	0.00003
8	− 1.02	36.565	0.092	79.26	19.71	0.94	0.0006	0.00009
9	− 1.051	36.518	0.087	79.31	19.64	0.97	0.0004	0.00005
10	− 1.027	36.503	0.081	78.69	20.16	1.07	0.0003	0.00005
11	− 1.016	36.507	0.073	79.14	19.76	1.03	0.0004	0.00005
12	− 1.02	36.494	0.073	79.16	19.82	0.95	0.0005	0.00006
13	− 1.04	36.56	0.075	79.11	19.85	0.97	0.0005	0.00003
14	− 1.053	36.559	0.074	79.15	19.83	0.95	0.0004	0.00004
15	− 1.127	36.481	0.075	79.28	19.70	0.94	0.0006	0.00004
16	− 1.197	36.374	0.075	78.43	20.57	0.92	0.0019	0.00001
17	− 1.115	36.383	0.081	78.18	20.81	0.93	0.0040	0.00002
18	− 1.161	36.387	0.075	78.59	20.39	0.94	0.0015	0.00001
19	− 1.17	36.383	0.076	78.44	20.56	0.93	0.0012	0.00001
20	− 1.164	36.387	0.078	78.35	20.64	0.93	0.0007	0.00001
21	− 1.178	36.374	0.084	78.19	20.78	0.93	0.0015	0.00002
22	− 0.86	36.283	0.078	78.44	20.54	0.94	0.0029	0.00002
23	− 0.854	36.278	0.080	78.33	20.65	0.93	0.0018	0.00002
24	− 0.85	36.284	0.079	78.20	20.79	0.93	0.0047	0.00002
25	− 0.853	36.283	0.078	78.47	20.52	0.93	0.0005	0.00004

**Figure 5:**
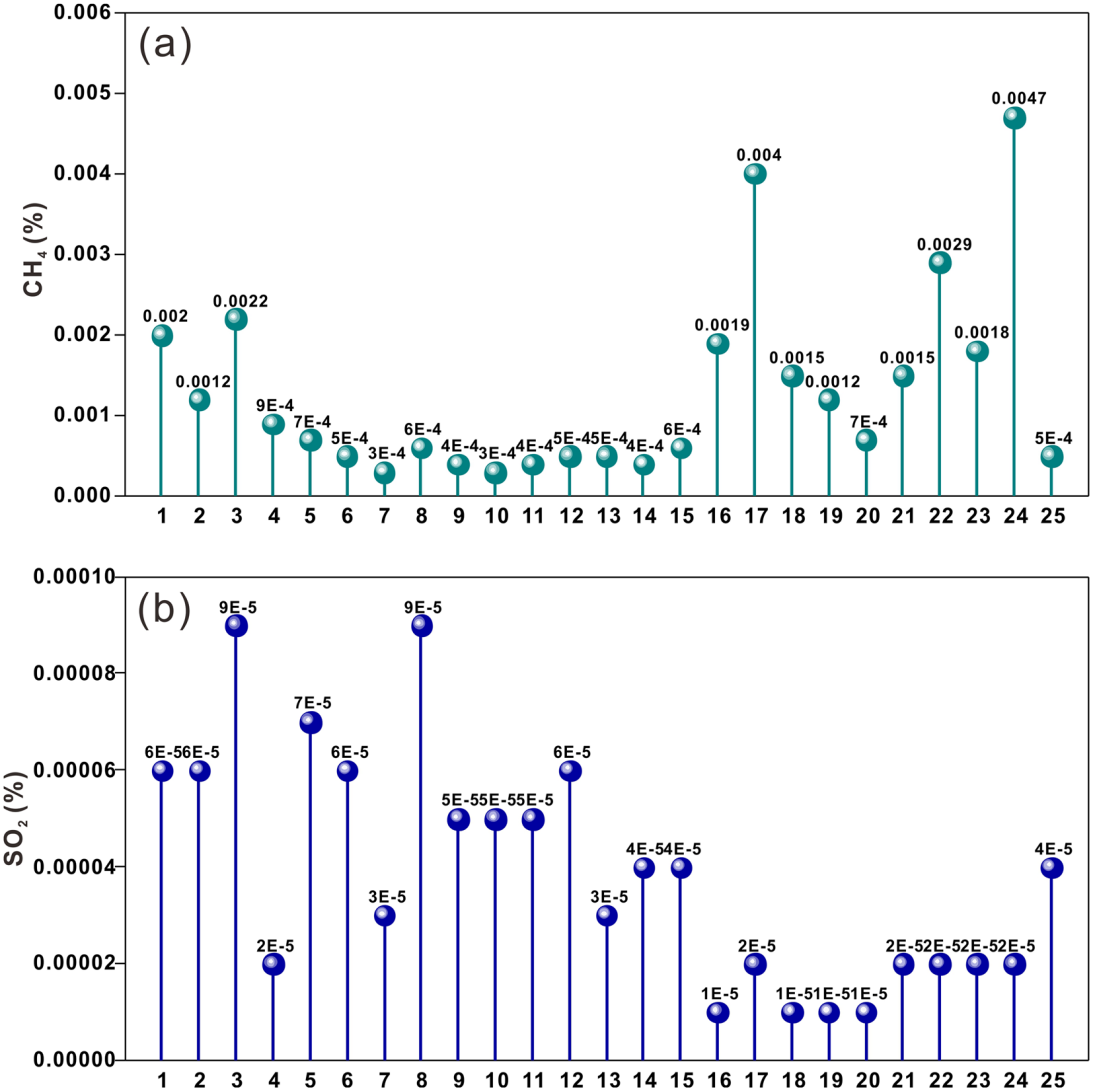
Trace gas composition characteristics of (**a**) methane and (**b**) sulfur dioxide. Gases near the crater of Mt. Suswa and Olkria have higher concentrations of CH_4_ relative to other regions, while gases near the eastern rift margin boundary faults has higher concentrations of H_2_.

The main trace components in the gas samples were methane (CH_4_) and sulfur dioxide (SO_2_). CH_4_ and SO_2_ were detected in every sample, and their abundances were up to 0.0047 and 0.0009%, respectively. As CH_4_ is directly derived from mantle magma and easily migrates upwards, it is generally regarded as an important trace component in magma source gas^85,86^. The detected CH_4_ indicates the deep mantle source of the gas^87^. Sulfur-containing gas is the main gas component in the active magma chamber^88^; although the SO_2_ concentration of the gas samples is low, it still differs from that in the atmosphere. Figure 5a shows that samples 17 and 24, located on the volcanic rock platform, have higher methane concentrations, which is likely due to volatile components in the magma chamber more easily entering the rock fissures close to the crater of Mt. Suswa and Olkria. Figure 5b shows that the gas escaping the rock fractures on both sides of the fault has a higher abundance of SO_2_ (as observed in samples 1, 2, and 8). This suggests that the rift margin boundary faults in the study area may provide additional channels for the transport and discharge of deeply sourced gas components into rock fractures.

Helium (He) has two isotopes, ^4^He and ^3^He, as isotopic tracers that can provide information about the degassing history of the mantle, formation of the atmosphere, and mixing relationships between different mantle reservoirs^89^. As atmospheric He is universally used as a mass spectrometric standard, ^3^He/^4^He ratios in unknown samples relative to the atmospheric ratio could easily be expressed as R_unknown_/R_air_ (R/R_A_), wherein the atmospheric ratio is 1.40 × 10^−6^^90^. Air-corrected ^3^He/^4^He values (R_c_/R_A_) were used in this study; that is, the ^3^He/^4^He ratio represents the sum of only two components: the ^3^He/^4^He ratios in the crust and mantle, respectively (see “Methods” section).

**Figure 6:**
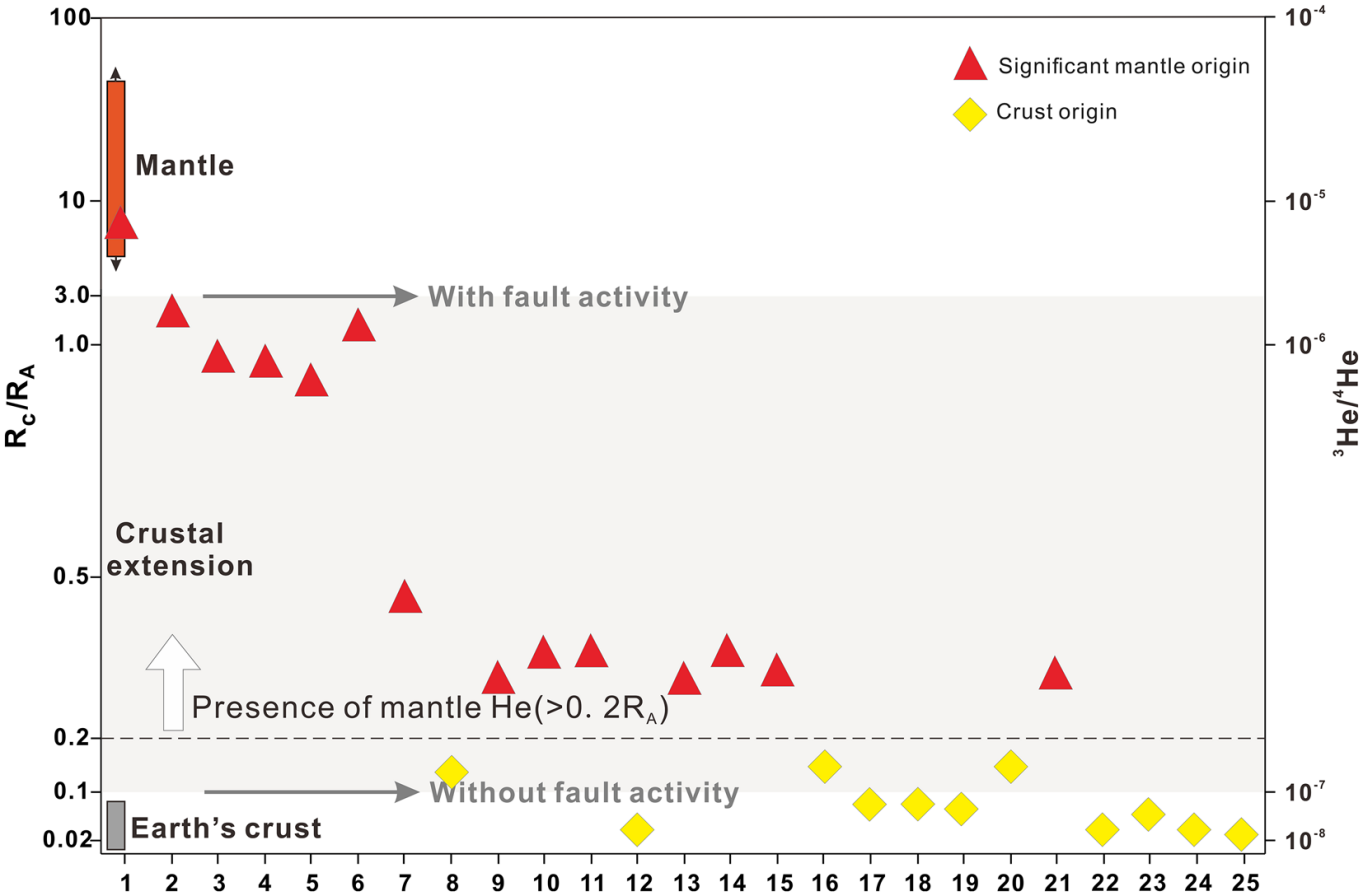
Plot of the R_c_/R_A_ ratios: (R/R_A_)_crust_ = 0.02; (R/R_A_)_crust extension without shear_ = 0.1; (R/R_A_)_crust extension with shear_ = 3; and (R/R_A_)_MORB_ = 7.2. The orange and gray bars represent the mantle and crust members^89^, respectively; the gray shaded area represents crustal extension, and the gray arrows represent the R/R_A_ value with and without fault activity, respectively^92^.

The R_c_/R_A_ values of the samples are shown in Table 2. The R_c_/R_A_ ratios of the gas samples range from 7.4 to 0.01. An enrichment in ^3^He/^4^He (i.e., > 0.20 R_A_) is considered to indicate an unequivocal contribution of mantle He in the source region^91^. The available He isotope data indicate that the R_c_/R_A_ values in more than half of the samples are > 0.2 (~ 10 times the typical crustal ^3^He/^4^He value). Furthermore, the R_c_/R_A_ ratio of sample No. 1 (7.4) is consistent with that of the mantle, showing that the rock fracture is very deep and may directly reach the mantle or the magma chamber in the crust. When there is normal fault activity in the upper crust, the R/R_A_ value of the Earth’s surface can reach as high as 3; the value is ~ 0.1 when there is no fault activity^92^. Figure 6 shows that the crust in the study area is not only in an extensional state but also has active faulting, which provides additional evidence for connections between the deep crust to mantle and the surface.

**Table 2:** ^3^He/^4^He data and the values of R_C_/R_A_, the location of gas samples are shown in Table 1.

Number	3He/4He	R_C_/R_A_
1	1.46E−06	7.4
2	1.50E−06	1.71
3	1.37E−06	0.88
4	1.37E−06	0.83
5	1.32E−06	0.75
6	1.35E−06	1.67
7	1.18E−06	0.42
8	1.13E−06	0.12
9	1.02E−06	0.28
10	1.02E−06	0.34
11	9.88E−07	0.34
12	9.85E−07	0.03
13	9.70E−07	0.27
14	9.88E−07	0.34
15	9.80E−07	0.29
16	9.82E−07	0.15
17	1.07E−06	0.07
18	1.06E−06	0.08
19	9.87E−07	0.05
20	1.01E−06	0.15
21	9.45E−07	0.27
22	1.05E−06	0.03
23	9.79E−07	0.07
24	9.17E−07	0.07
25	9.58E−07	0.01

Most of the escaping gas within the fissures had a mantle contribution (red triangles in Fig. 6), indicating the deep origin of the fissure. This shows that most rock fractures that extended below the ground fissures were relatively deep rather than shallow. The rock fractures may be connected to the magma chamber of the crust, upper mantle, or branches of the deep faults. The samples with an R_c_/R_A_ ratio below 0.2 (yellow diamonds in Fig. 6) cannot prove the deep location of fractures. However, we demonstrated that these ground fissures have considerably deep origins by characterizing the composition of gases escaping the fractures, including CO_2_ quantities, trace gas quantities, and He isotopes. Considering the contribution of the mantle in the gas samples, the ground fissures may originate from the crust at a depth of more than ten kilometers, and may even originate from the Moho surface at a depth of tens of kilometers.

### Geodetic strain and rock fractures

**Figure 7:**
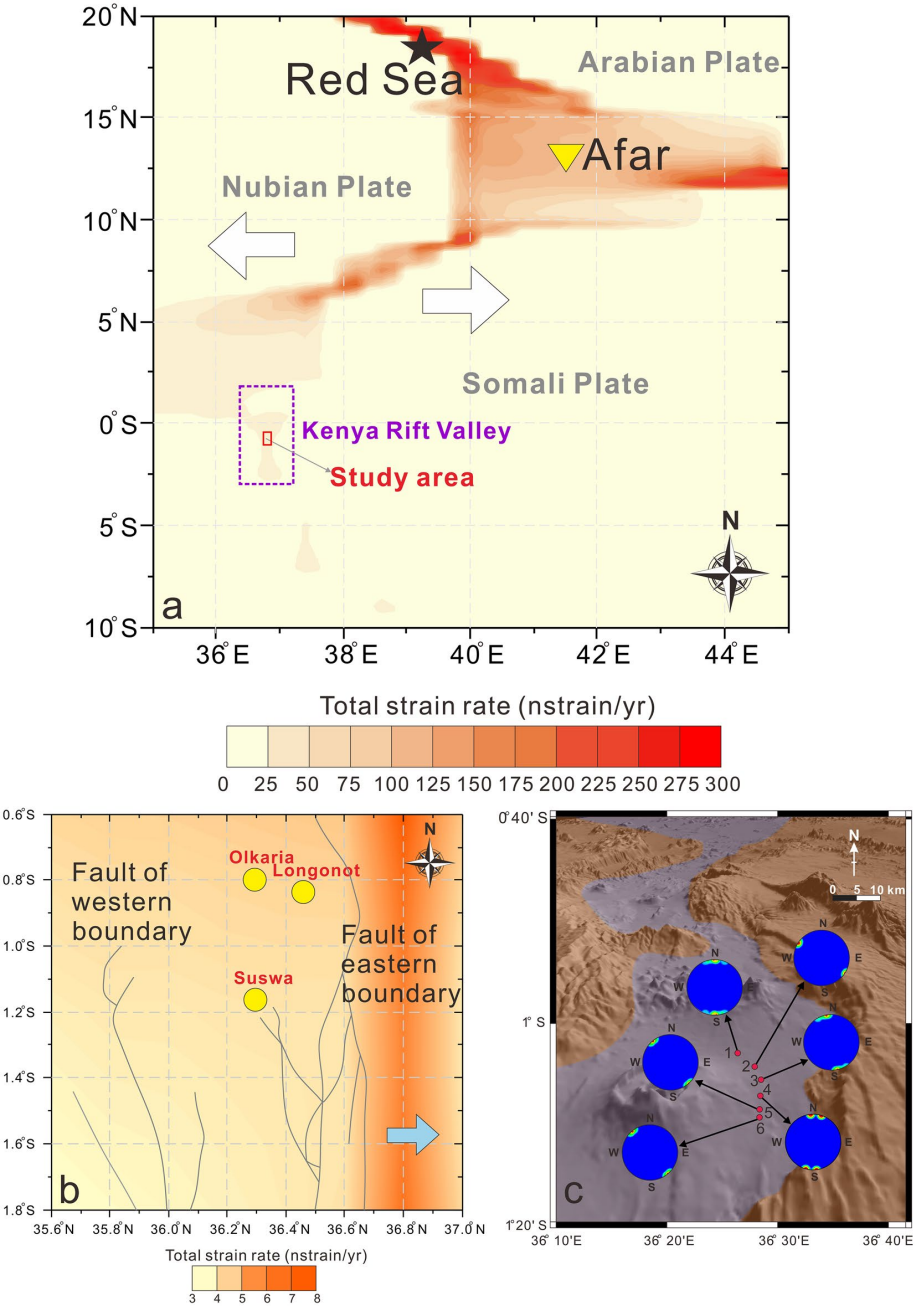
(**a**) Contour map of the geodetic strain-rate field of the Great Rift Valley based on the Global Strain Rate Model^93^. The black star represents the red sea; the yellow triangle represents the Afar triple point; and the white arrows point in the direction of lithospheric detachment. (**b**) Contour map of the geodetic strain-rate field of the study area based on the Global Strain Rate Model. Gray solid lines represent faults; yellow solid circles represent volcanoes; and the blue arrow points in the direction of lithospheric detachment. (**c**) Distribution of strikes of rock fractures in the study area. Red solid circles and numbers represent the field survey points, and the blue solid circles represent the statistical diagram of the strike of rock fractures.

Owing to the strong plate divergence in the Great Rift Valley, the influence of the regional stress state on the crustal rock fracture system should be considered. Figure 7a shows the geodetic strain-rate field in the Great Rift Valley. The Great Rift Valley generally extends in an east–west direction, and the strain rate at the northern end of the rift is significantly higher than that in the south. In southern Kenya, the strain was negligible. Figure 7b shows the geodetic strain-rate field in the study area. The eastern boundary fault zone exhibits the highest strain rate. This imbalance in strain distribution may explain why there are more ground fissures on the east side of the study area than on the west side. The fractures on the eastern side of the study area have a higher activity rate, which further aggravates the deformation of the solid crust and generates fissures. In Fig. 7c, we compiled the measured strikes of rock fractures exposed in the valley. The results show that the strike of most of these rock fractures is in the NNW–SSE direction. The strike of rock fractures in a rift environment is perpendicular to the tensile stress direction, which indicates that crustal deformation caused by plate divergence is one of the causes of the rock fractures. These rock fractures intersect with each other and may be connected to faults, magma chambers, and geothermal fluids. The response of deep rock fractures to plate divergence results in the final opening of ground fissures at the surface.

## Discussion

**Figure 8:**
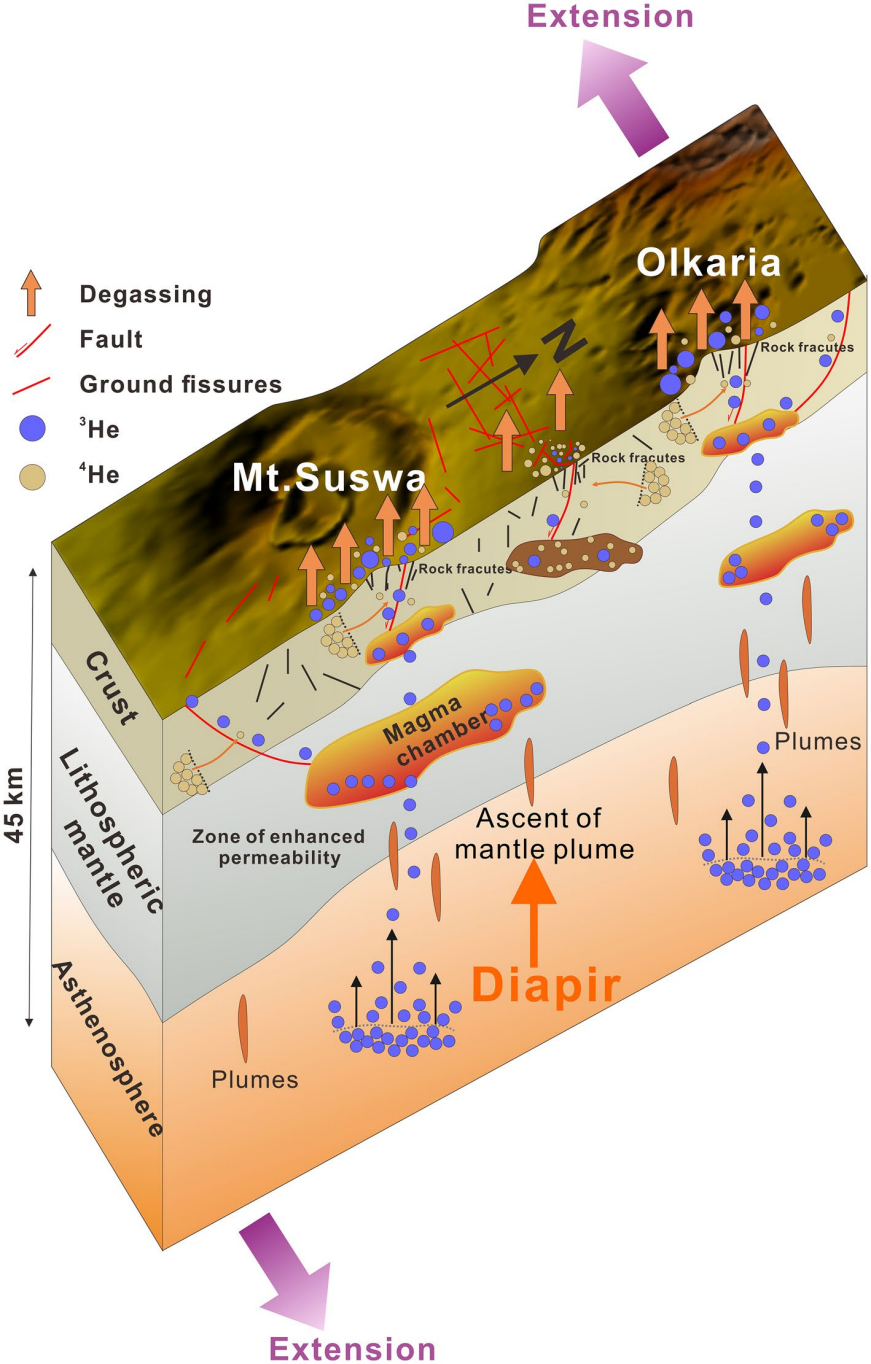
Interpreted formation mechanism of ground fissures in the Kenya Rift Valley. The selected profiles are Mt. Suswa and Olkaria volcano and the rift valley floor between them. The orange upward arrow represents the asthenosphere diapir; the orange strips represent small plumes of magma derived from the asthenosphere; the orange-crimson polygons represent hot magma chambers; the brown polygons represent cooled magma chambers; the short black lines represent the rock fractures in the crust; and the purple arrows represent the direction of plate divergence.

Our results demonstrate that ground fissures in loose sediments are connected to deep underlying rock fractures in volcanic rocks (Figs. 3, 4 and 6), and that these deep rock fractures may have been formed by several geological processes such plate divergence and volcanic activity. Figure 8 shows the formation mechanism of ground fissures with deep origins. Owing to the widespread asthenosphere diapir and lithospheric scale destruction in the Great Rift Valley^94^, rifting and volcanic activity may have caused a wide range of rock ruptures. The asthenospheric diapir can lead to the formation of many fractures in the lithospheric mantle and impact the homogeneity of the lithosphere. The samples falling into the gray shaded area in Fig. 6 may indicate that some fractures are not directly connected to the mantle or volcanic magma chamber, whereas the R_C_/R_A_ values between 0.1 and 3 indicate the existence of the zone of enhanced permeability in plastic lower crust. The zone of enhanced permeability formed by the fractures is not only helpful for the injection of mantle magma into the crust, but also provides advantageous channels for the migration of deep mantle volatiles^92^. These fractures in the mantle lithosphere may extend all the way up to the pyroclastic roof below the sediments. The rock fractures of this origin generally have the greatest depth, and the gas they carry may contain directly detect mantle-derived He. The R_C_/R_A_ value (7.4) of gas sample No. 1 is consistent with that of the mantle (Fig. 6), which may reveal a fracture connecting the volcanic magma chamber, and even this fault may extend all the way down to the lower crust.

The origin of another type of ground fissure was related to faults. Boundary faults that control the morphology of the rift are often associated with initial active rifting and plate divergence^95^. The expansion of the volcanic magma chambers commonly forms annular rock fractures and faults around volcanic craters in the crust^65^. The generation of these fractures and faults is unaffected by the accumulation of tensile stress caused by plate divergence, but depends mainly on the expansion of the magma chamber^96^. Fault degassing is common in a fault channel with high permeability and porosity^97^. When the faults are connected to the magma chamber, mantle volatiles (^3^He) in the magma chamber migrate to the surface through the fault; thus, the gases expelled from the fault contain evidence for mantle contributions. Some He from the crust in the gas samples in this study may be related to the cooling of the magma chambers in the crust. The R_C_/R_A_ values of gas samples No. 12 and No. 22–24 shown in Fig. 6 are consistent with that of the crust (~ 0.02), and these samples have high CH_4_ quantities (Fig. 5). This feature may indicate that the volatile substances of these gas samples come from cooling volcanoes magma chambers. These cooled magma chambers are no longer actively supplied by magma, and their residual volatiles are only diluted by crustal helium; consequently, they mainly contain components from the crust when they reach the surface along rock fractures.

There are few cases of helium isotopes from gas in Kenya Rift. Hilton et al.^98^ reported a case of helium isotope ratios of lavas and tephra from the Rungwe Volcanic Province (RVP) in southern Tanzania. The RVP is located in the southernmost part of the Kenya Rift, and its ^3^He/^4^He ratios of lavas and tephra are consistent with that of the lower mantle. Our data from gas samples also indicate the mantle origin of these ^3^He/^4^He ratios, and together with the helium isotope cases in the southern and northern Great Rift Valley, support the existence of the African Superplumes^99^. Hilton et al. also suggested that these high ^3^He/^4^He signals were transferred to the surface through rift‐related volcanism, which was elaborated in our initial discussion about the origin of ground fissures.

Faults or rock fractures associated with deep activity exist as ground fissure prototypes in the crust. As the plate diverges, a large amount of tensile strain accumulates in the crust, and such tensile strain can lead to further opening and extension of faults or fractures to release the accumulated tensile stress^96^. Our field mapping results indicate that these rock fractures may open in the direction perpendicular to the minimum principal tensile stress (Fig. 7c). The divergence of the plates on both sides of the rift expands the size of the rock fractures, make the width of the typical rock fractures to 15 cm (Fig. 3b,d) and enables them to extend upwards to the shallow sedimentary cover. We suggest that occurrence of the ground fissures in the study area, is mainly controlled by rock fractures at different depths and widths and triggered by surface deformation or erosion.

Chatterjee et al. (2023) studied the first ground fissure reported in the Central Kenya Rift by differential interferometric SAR method and GNSS data, and they believed that dyke intrusion along the pre-existing fault and reactivation of the fault were the primary factor for the formation of ground fissures^100^. Effective stress reduction of sediments after heavy raninfall and erosion are triggering factor for ground fissure formation. Our field surveys support piping-related erosion and reduction in sediment strength as triggering factor for ground fissure formation (Fig. 2). However, in addition to the ground fissure in Mai Mahiu town reported in 2018, we also found that similar ground fissures are widespread in the study area, but their scale is smaller than that of Mai Mahiu town. After comparing the data set of Chatterjee et al. (2023) on surface deformation with our ground fissure distribution map (Fig. 1a), we found that there is no dense distribution of ground fissures near Mt. Suswa and Mt. Longonot, which are the two volcanoes with the most intense surface deformation today. This seems difficult to explain by dyke intrusion related to volcanic activity. Most of the ground fissures are concentrated on both sides of the inner rift, especially near the eastern boundary fault which is in good agreement with the high strain rate area (Fig. 7b), may imply effect of the fault reactivation related to plate extension on ground fissures^42^. It seems reasonable to explain the formation of the Mai Mahiu giant ground fissures by episodic magmatic inflation, volcanism and dyke intrusion, but it is difficult to explain such a wide distribution of ground fissures in the study area. A more likely reason is that the rock fractures that are the prototype for ground fissures were formed by multiple volcanic and faulting events over a long period of time, and these fractures were subsequently covered or filled by sediments. Effect of rock fractures on ground fissures mainly occurs through the surface processes. Unconsolidated sediments are continuously lost into the rock fractures, and weak zones along the fractures are formed in the sediments. The weak zones are easily widened through erosion by the surface runoff or piping, which produces large ground fissures. This may explain the reported occurrence of ground fissures actively forming in the KRV after heavy rain^53^.

## Conclusion

Through field investigation, trenching, geophysical exploration, and sample analysis, we identified the deep origins of 22 ground fissures in the KRV for the first time. Ground fissures in the KRV are mainly hazardous to the safety of roads, culverts, railways, and communities. Ground fissures continually damage the foundations of buildings and eventually lead to their failure. Trenching and geophysical explorations show that the ground fissures in unconsolidated sediments are always connected to degassing rock fractures below, and that the ground fissures and rock fractures have a consistent strike. CO_2_ of gas samples account for approximately 0.03–0.09% of the total composition, more than twice that of the atmosphere, which indicates the difference between these gases and the normal atmosphere. CH_4_ and SO_2_ are detected in all gases escaping the rock fractures, indicating the deep mantle origin. Furthermore, through the analysis of Helium isotopes, we show that the R_C_/R_A_ ratio of most gas samples is greater than 0.2, and the maximum can reach 7.4, which indicates most of the gas escaping through ground fissures and rock fractures were derived from the mantle. Rock fractures and faults are prototypes of ground fissures, and confirm their deep origin. The eastern boundary fault zone exhibits the highest strain rate. Strike of most of these rock fractures is in the NNW–SSE direction, which is perpendicular to the direction of plate movement. Rock fractures with gas escaping are formed due to plate divergence, volcanic activity, then the ground fissures develop due to surface processes. After determining the deep origin of the ground fissures, recommendations can be made for preventing ground fissure disasters in this and other areas in the rift valley. First, extensive geological surveys are necessary to identify the distribution of rock fractures beneath sediments. Second, extreme weather, especially heavy rain, should be monitored as a potential cause for ground collapses due to the rapid development of ground fissures at the surface. We believe that this study can not only assist in engineering siting and urban planning in this region and other areas with similar geological conditions, but can also contribute to improving the safety of local communities.

## Methods

### Field investigation and trenching

This study focuses on the Kedong Basin in the Central Kenya Rift (see Fig. 1), which is a part of the east branch of the Great Rift Valley of East Africa. The locations of ground fissures and gas samples in the entire study area were determined through field investigations and GPS positioning. The length, width and depth and strike of these ground fissures were measured by range finder, steel tape and compass, respectively. We used excavators to dig three trenches to reveal rock fractures below the ground fissures. The locations of the three trenches are shown in Fig. 1b. Surface shape of each trench is a rectangle of 20 × 18 m. These three trenches cut through all the sediments and reach the underlying pyroclastic rocks, so their depths vary, trench No. 1 is 6.5 m deep, trench No. 2 is 8 m deep, and trench No. 3 is 4.8 m deep. The long axis of trench is perpendicular to the strike of the ground fissure.

### Geophysical exploration

We used a WGMD-6 electrical apparatus for determining resistivity of crust at a depth of 0–40 m. Considering factors such as the resolution and detection depth, an electrode distance of 5 m was selected for data acquisition. The electrical apparatus is powered by direct current with the 300–600 V input voltage, and the copper electrodes were sued to minimize ground resistance. We used a SWS seismometer with 12 receiving channels for the shallow seismic refraction method to determine the seismic wave of crust at a depth of 0–40 m. The source type is hammer excitation, the channel spacing is 2 m, the offset distance is 20 m, and the coverage is sixfold.

### Sampling and laboratory analyses

All the gas samples were collected using a TQC-1500 gas sampler and Fluorinated ethylene propylene (FEP) gas sampling bag. The FEP sampling bag with airtight valve has the characteristics of solvent corrosion resistance, heat resistance, aging resistance and radiation resistance, and is ideal for storing various gaseous samples with strong volatility, corrosion and high chemical activity. To eliminate air pollution as much as possible, we run the gas sampler without connecting the sampling bag and sampling for 1 min to empty the residual gas in the sampler; then we connect the pre-vacuum sampling bag to the sampler and start sampling. After sampling, the valve of the sample bag was closed and sealed by aluminum foil, then the sampler was closed. The gas samples were transported from Kenya to China by ocean shipping and analyzed a month after sampling. Gas samples were analyzed at the Key Laboratory of Petroleum Resources Research, Chinese Academy of Science, Lanzhou, China. The abundances of CO_2_, N_2_, O_2_, and Ar were determined using a PrismaPlus mass spectrometer and gas chromatograph (GC9560). The ^3^He/^4^He and ^4^He/^20^Ne ratios were determined using a Noblesse mass spectrometer (Noblesse).

### Data processing

Air-corrected ^3^He/^4^He values (R_C_/R_A_) were used to correct any measured He isotopic compositions for air-derived contributions, and the air-corrected ^3^He/^4^He value was calculated using the following equation^101^:

1 $$R_{C} /R_{A} = \left[ {(R/R_{A} \times X) - 1} \right]/\left( {X - 1} \right),$$

2 $$X = \left[ {\left( {{}_{ }^{4} He/{}_{ }^{20} Ne} \right) _{measured} /\left( {{}_{ }^{4} He/{}_{ }^{20} Ne} \right) _{air} } \right],$$

3 $$\left( {{}_{ }^{4} He/{}_{ }^{20} Ne} \right) _{air} = 0.318.$$

## Supplementary Information

Supplementary Information.

## Data Availability

All data generated or analysed during this study are included in this published article and its supplementary information files.
